# Supplementary material for: A Comparative Analysis of Influenza-Associated Disease Burden with Different Influenza Vaccination Strategies for the Elderly Population in South Korea
Source: Vaccines (Basel). 2022 Aug 25;10(9):1387. doi: 10.3390/vaccines10091387 (PMC9503807; doi:10.3390/vaccines10091387)
Supplement: Supplementary file 1 [file vaccines-10-01387-s001.zip › vaccines-1838451-supplementary.pdf]

Supplementary Materials for:

A Comparative Analysis of Influenza-Associated Disease Burden with Different Influenza Vaccination Strategies for the Elderly Population in South Korea

Min Joo Choi [1], Jae-Won Yun [2], Joon Young Song [2,3], Karam Ko [4], Joaquin F. Mould [5] and Hee Jin Cheong [2,3,*]

[1] Department of Internal Medicine, International St. Mary's Hospital, Catholic Kwandong University College of Medicine, Incheon 22711, Korea
[2] Asia Pacific Influenza Institute, Korea University College of Medicine, Seoul 08308, Korea
[3] Department of Internal Medicine, Guro Hospital, Korea University College of Medicine, Seoul 08308, Korea
[4] Seqirus Korea Ltd., Seoul 03157, Korea
[5] Seqirus USA Inc., Summit, NJ 07901, USA
* Correspondence: heejinmd@korea.ac.kr

Table S1: Sensitivity analysis parameter

Parameter name (range)	Base-case values	Lower Limit	Upper limit	Type of Distribution
Influenza infection incidence rate among low-risk unvaccinated 65y+	7.2%	4.3%	12.0%	Beta
Probability of hospitalization for low-risk without complication in 65-74y	0.4%	0.4%	0.4%	Beta
Probability of hospitalization for low-risk without complication in 75-84y	0.5%	0.4%	0.5%	Beta
Probability of hospitalization for low-risk without complication in ≥85y	0.8%	0.7%	0.9%	Beta
Probability of hospitalization for low-risk with complication in 65-74y	4.8%	4.3%	5.3%	Beta
Probability of hospitalization for low-risk with complication in 75-84y	8.9%	8.0%	9.8%	Beta
Probability of hospitalization for low-risk with complication in ≥85y	15.2%	13.7%	16.7%	Beta
Probability of hospitalization for high-risk without complication in 65-74y	1.8%	1.6%	2.0%	Beta
Probability of hospitalization for high-risk without complication in 75-84y	2.3%	2.1%	2.6%	Beta
Probability of hospitalization for high-risk without complication in ≥85y	3.9%	3.5%	4.3%	Beta
Probability of hospitalization for high-risk with complication in 65-74y	43.6%	39.2%	47.9%	Beta

Probability of hospitalization for high-risk with complication in 75-84y	56.2%	50.6%	61.8%	Beta
Probability of hospitalization for high-risk with complication in ≥85y	65.6%	59.1%	72.2%	Beta
QIV vaccine effectiveness against H1N1 in the Elderly population	62.0%	36.0%	79.0%	Normal
QIV vaccine effectiveness against H3N2 in the Elderly population	24.0%	0.0%	45.0%	Normal
QIV vaccine effectiveness against B in the Elderly population	63.0%	33.0%	79.0%	Normal
aQIV vs QIV relative effectiveness	13.9%	4.2%	23.5%	Normal
aQIV vs HD-QIV relative effectiveness	3.2%	-2.5%	8.9%	Normal
Vaccination coverage 65–74 years population	80.0%	72.0%	100.0%	Beta
Vaccination coverage 75–84 years population	80.0%	72.0%	100.0%	Beta
Vaccination coverage 85+ years population	80.0%	72.0%	100.0%	Beta

Parameter name (range)	Base-case values	Lower Limit	Upper limit	Type of Distribution
Probability of comorbidities for 65–74 years population in Korea	16.6%	14.9%	18.3%	Beta
Probability of comorbidities for 75–84 years population in Korea	23.6%	21.2%	26.0%	Beta
Probability of comorbidities for 85+ years population in Korea	25.7%	23.1%	28.3%	Beta
Probability of death for low-risk inpatient without complication in 65-74y	0.3%	0.2%	0.3%	Beta
Probability of death for low-risk inpatient without complication in 75-84y	0.3%	0.3%	0.4%	Beta
Probability of death for low-risk inpatient without complication in ≥85y	1.8%	1.6%	1.9%	Beta
Probability of death for low-risk inpatient with complication in 65-74y	1.3%	1.2%	1.4%	Beta
Probability of death for low-risk inpatient with complication in 75-84y	2.1%	1.9%	2.3%	Beta
Probability of death for low-risk inpatient with complication in ≥85y	7.3%	6.6%	8.0%	Beta
Probability of death for high-risk inpatient without complication in 65-74y	2.4%	2.2%	2.6%	Beta
Probability of death for high-risk inpatient without complication in 75-84y	3.2%	2.9%	3.5%	Beta
Probability of death for high-risk inpatient without complication in ≥85y	5.9%	5.3%	6.5%	Beta

Probability of death for high-risk inpatient with complication in 65-74y	4.0%	3.6%	4.4%	Beta
Probability of death for high-risk inpatient with complication in 75-84y	7.2%	6.5%	7.9%	Beta
Probability of death for high-risk inpatient with complication in ≥85y	16.0%	14.4%	17.5%	Beta
Probability of death for low-risk outpatient without complication in 65-74y	0.0%	0.0%	0.0%	Beta
Probability of death for low-risk outpatient without complication in 75-84y	0.1%	0.1%	0.1%	Beta
Probability of death for low-risk outpatient without complication in ≥85y	0.6%	0.6%	0.7%	Beta
Probability of death for low-risk outpatient with complication in 65-74y	0.1%	0.1%	0.1%	Beta
Probability of death for low-risk outpatient with complication in 75-84y	0.2%	0.2%	0.3%	Beta
Probability of death for low-risk outpatient with complication in ≥85y	1.7%	1.5%	1.9%	Beta
Probability of death for high-risk outpatient without complication in 65-74y	0.4%	0.3%	0.4%	Beta

Parameter name (range)	Base-case values	Lower Limit	Upper limit	Type of Distribution
Probability of death for high-risk outpatient without complication in 75-84y	1.1%	1.0%	1.2%	Beta
Probability of death for high-risk outpatient without complication in ≥85y	3.3%	3.0%	3.7%	Beta
Probability of death for high-risk outpatient with complication in 65-74y	1.6%	1.5%	1.8%	Beta
Probability of death for high-risk outpatient with complication in 75-84y	3.3%	3.0%	3.7%	Beta
Probability of death for high-risk outpatient with complication in ≥85y	8.7%	7.9%	9.6%	Beta
Prevalence of influenza A (H3N2)	44.0%	20.9%	72.9%	Beta
Prevalence of influenza A (H1N1)	16.5%	0.5%	41.9%	Beta
Probability of complication for low-risk inpatient with influenza in 65-74y	3.2%	2.9%	3.5%	Beta
Probability of complication for low-risk inpatient with influenza in 75-84y	2.9%	2.6%	3.2%	Beta
Probability of complication for low-risk inpatient with influenza in ≥85y	4.0%	3.6%	4.4%	Beta
Probability of complication for high-risk inpatient with influenza in 65-74y	7.8%	7.0%	8.6%	Beta
Probability of complication for high-risk inpatient with influenza in 75-84y	10.3%	9.2%	11.3%	Beta

Probability of complication for high-risk inpatient with influenza in ≥85y	17.4%	15.7%	19.2%	Beta
Relative risk of influenza for patient with high-risk in 65-74y (1.0 - 1.34)	1.00	1.00	1.34	Normal
Relative risk of influenza for patient with high-risk in 75-84y	1.00	1.00	1.34	Normal
Relative risk of influenza for patient with high-risk in ≥85y	1.00	1.00	1.34	Normal

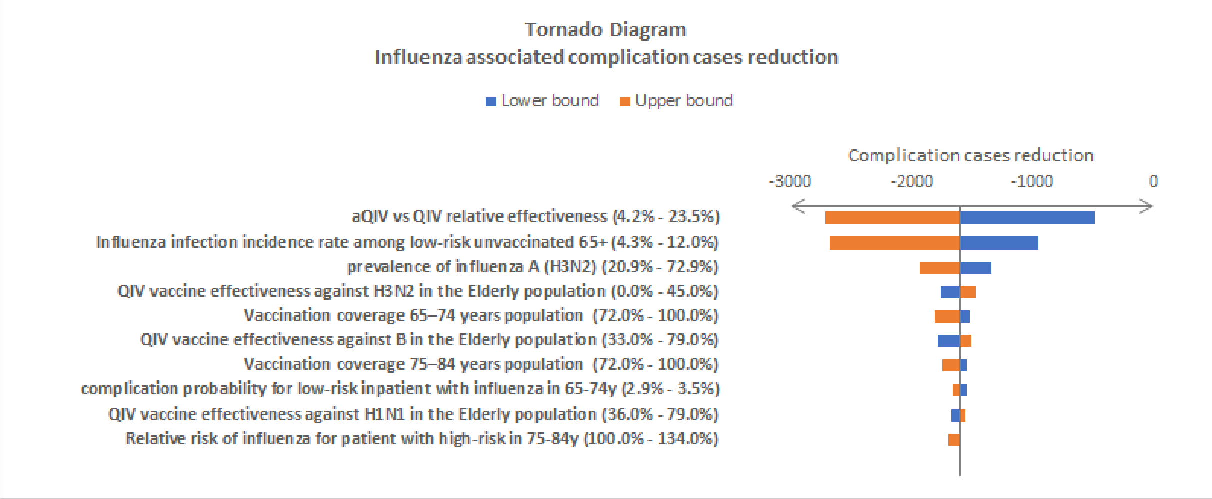

Figure S1: The top 10 most influential factors driving influenza associated complication case reduction in the comparison of using aQIV vs using QIV in NIP for the elderly in South Korea. The values in the parenthesis are the minimum and maximum values tested in the DSA.

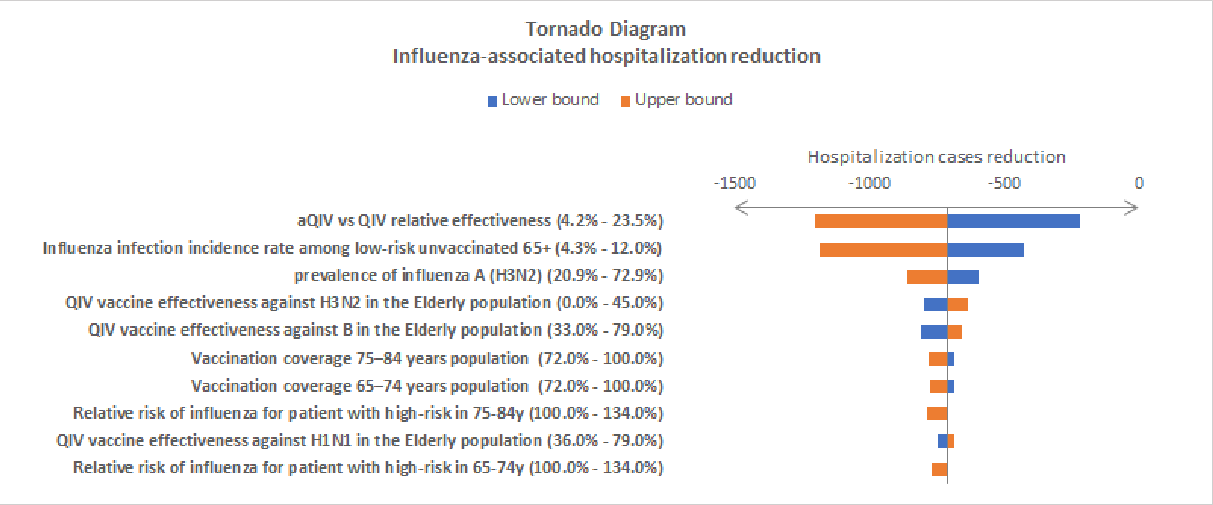

Figure S2: The top 10 most influential factors driving influenza associated hospitalization reduction in the comparison of using aQIV vs using QIV in NIP for the elderly in South Korea. The values in the parenthesis are the minimum and maximum values tested in the DSA.

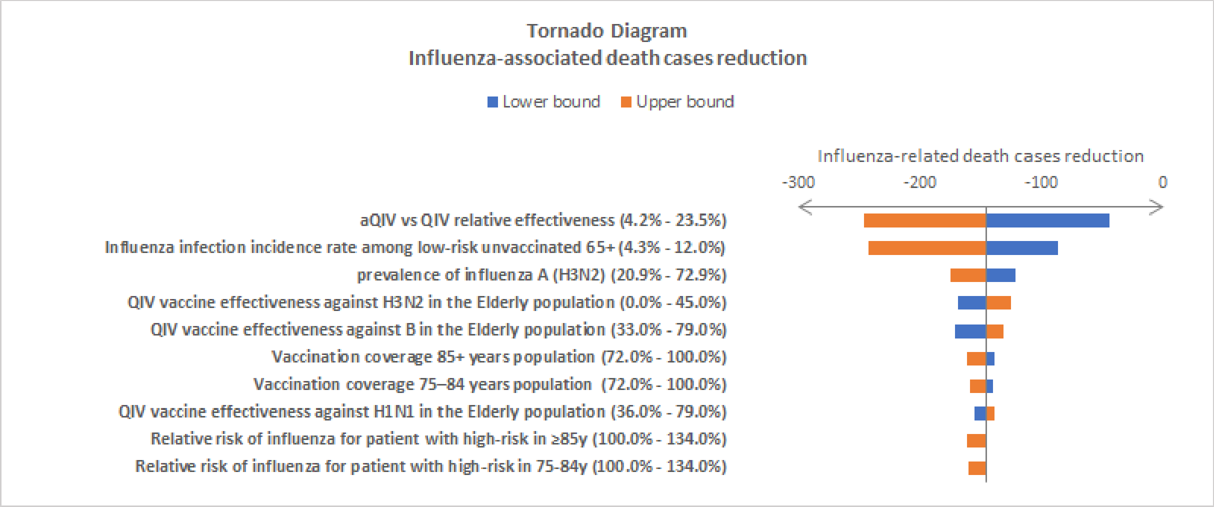

Figure S3: The top 10 most influential factors driving influenza associated death reduction in the comparison of using aQIV vs using QIV in NIP for the elderly in South Korea. The values in the parenthesis are the minimum and maximum values tested in the DSA.

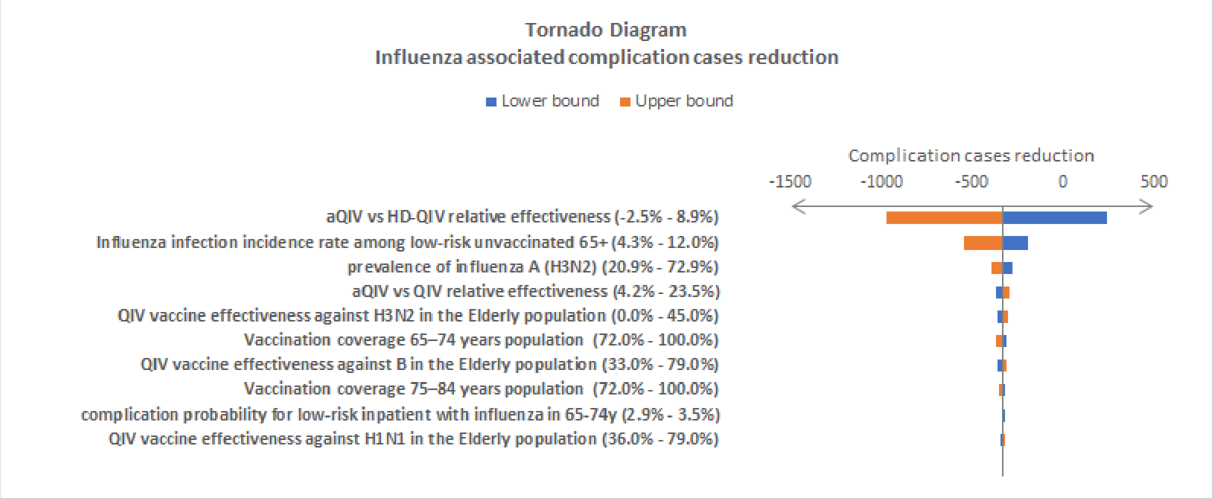

Figure S4: The top 10 most influential factors driving influenza associated complication reduction in the comparison of using aQIV vs using HD-QIV in NIP for the elderly in South Korea. The values in the parenthesis are the minimum and maximum values tested in the DSA.

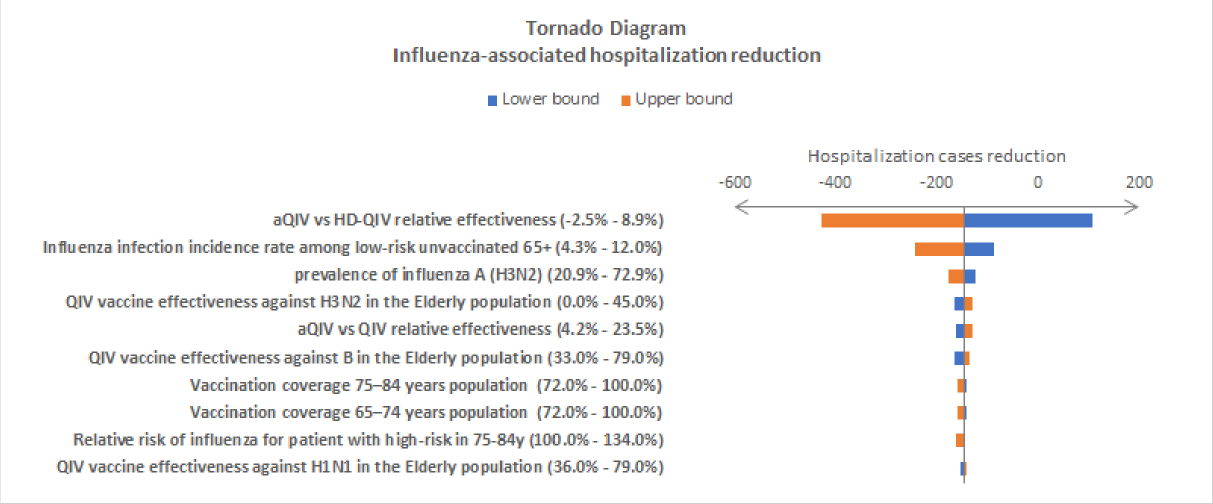

Figure S5: The top 10 most influential factors driving influenza associated hospitalization reduction in the comparison of using aQIV vs using HD-QIV in NIP for the elderly in South Korea. The values in the parenthesis are the minimum and maximum values tested in the DSA.

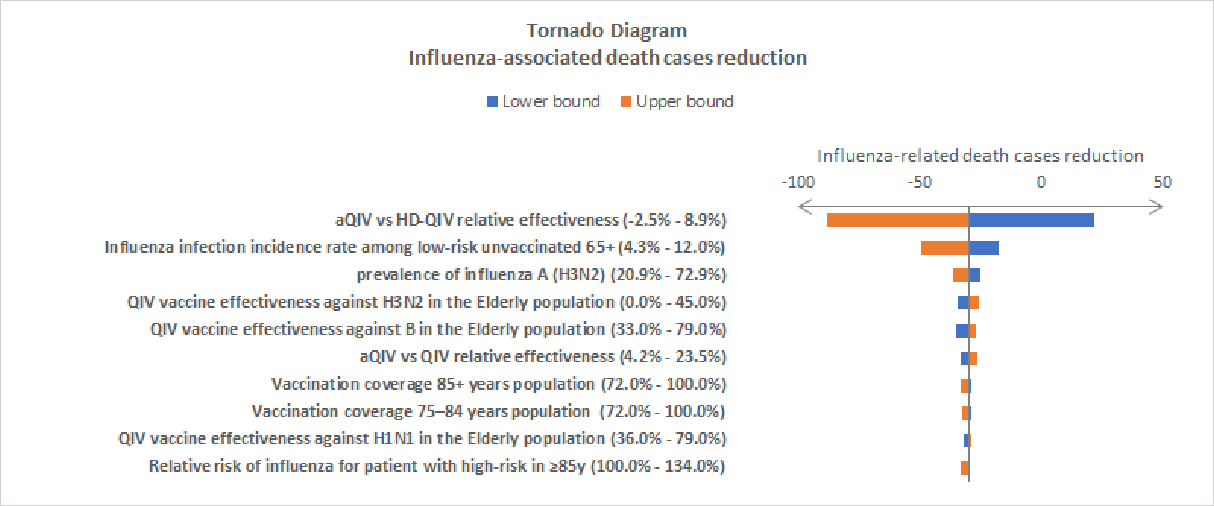

Figure S6: The top 10 most influential factors driving influenza associated death reduction in the comparison of using aQIV vs using HD-QIV in NIP for the elderly in South Korea. The values in the parenthesis are the minimum and maximum values tested in the DSA.

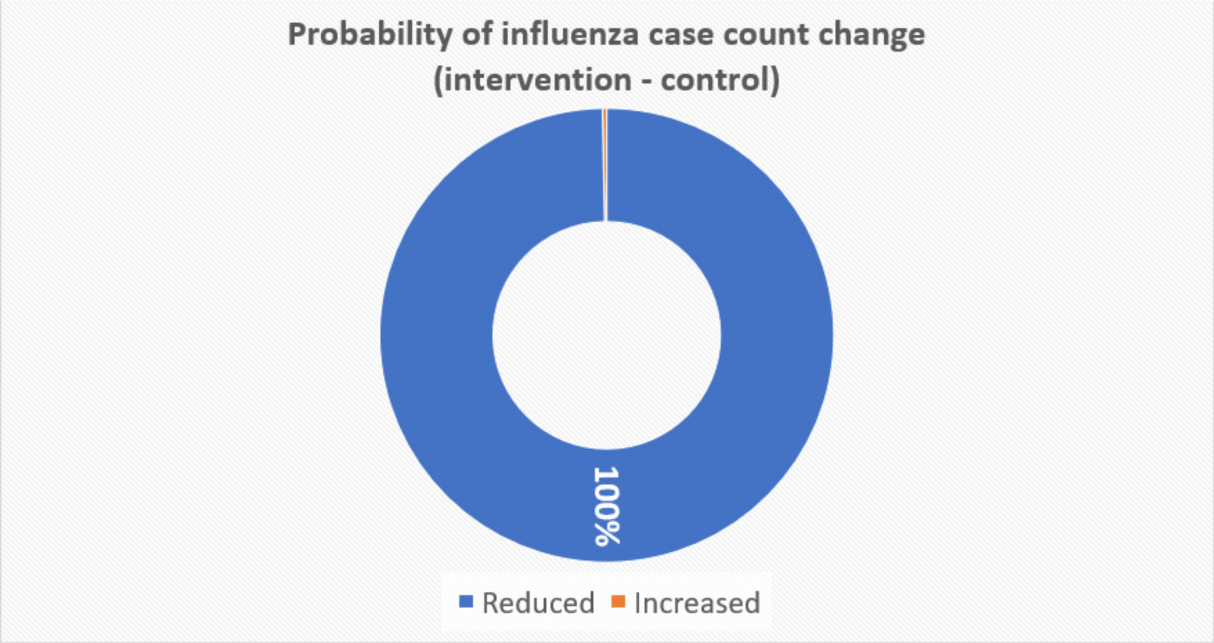

Figure S7: PSA case count comparison of using aQIV vs using QIV in NIP for the elderly population in South Korea.

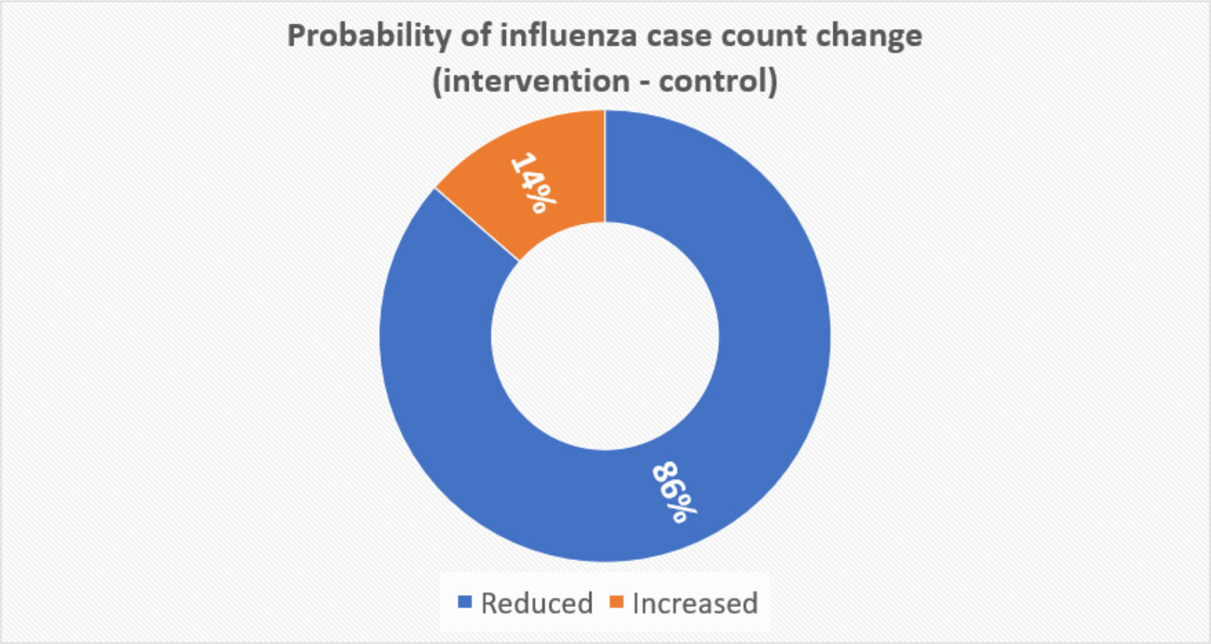

Figure S8: PSA case count comparison of using aQIV vs using HD-QIV in NIP for the elderly population in South Korea.

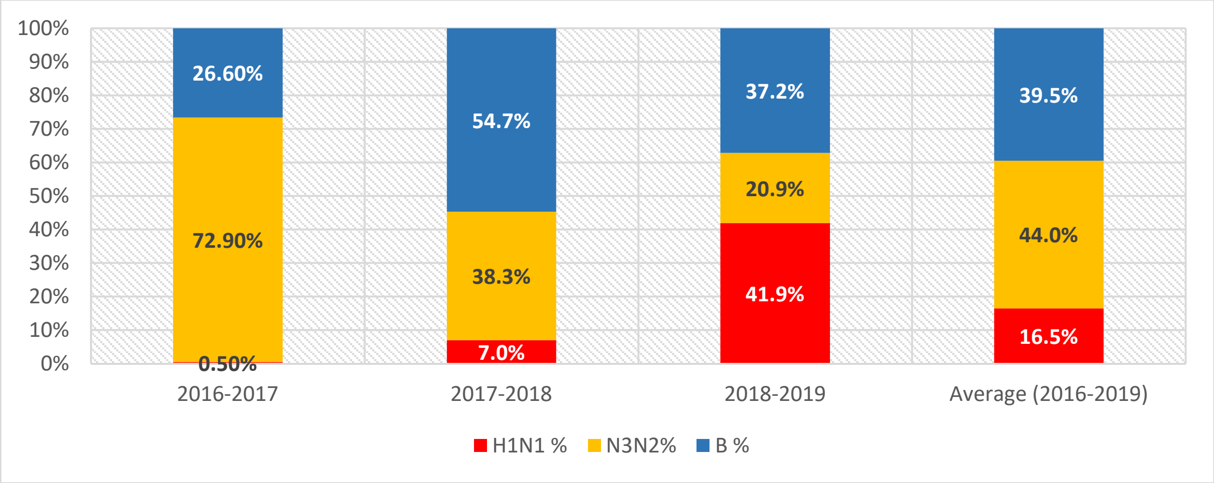

Figure S9. Influenza circulation in South Korea. Influenza surveillance data from the Korea Disease Control and Prevention Agency (KDCA) [19,20]
